#  Analysis of Aflatoxin B1 in Iranian Foods Using HPLC and a Monolithic Column and Estimation of its Dietary Intake 

**Published:** 2013

**Authors:** Hassan Yazdanpanah, Afshin Zarghi, Ali Reza Shafaati, Seyed Mohsen Foroutan, Farshid Aboul-Fathi, Arash Khoddam, Firoozeh Nazari, Fatemeh Shaki

**Affiliations:** a *School of Pharmacy, Shahid Beheshti University of Medical Sciences, Tehran, Iran.*; b *Noor Research and Educational Institute, Tehran, Iran.*

**Keywords:** Aflatoxin B1, Dietary intake, Foods, HPLC, Monolithic column

## Abstract

A high performance liquid chromatographic method was developed for determination of aflatoxin B1 (AFB1) in foods using a monolithic column with sample clean up on an immunoaffinity column. The method was validated for analysis of AFB1 in rice, bread, puffed corn snack, wheat flour and peanut samples. The average recoveries for AFB1 in different foods ranged from 94.4 to 102.5% with the coefficient of variation lower than 10% for all foods. Limit of detection was 0.01 ng/g. A survey of AFB1 was performed on 90 samples collected from Tehran retail market in June 2005. The results showed that none of the bread and wheat flour samples were contaminated with AFB1. The mean AFB1 levels in rice, puffed corn snack and peanut samples were 4.17, 0.11, and 1.97 ng/g, respectively. The level of contamination of 3 samples (one rice sample and two peanuts samples) to AFB1 was found to be higher than 5 ng/g. Although all food samples had mean concentration of AFB1 below the maximum tolerated level in Iran, the mean intake of AFB1 from rice was estimated 3.49 times higher than the guidance value of 1 ng AFB1/Kg body weight/day. Therefore, it is strongly recommended to monitor AFB1 in foods, especially in rice, in Iran. This is the first study on exposure assessment of Iranian population to AFB1.

## Introduction

Mycotoxins are secondary metabolites produced by microfungi that are capable of causing disease and death in humans and other animals (1). Aflatoxins (AFs) are one of the most important groups of mycotoxins which are considered to be economically and toxicologically important worldwide. AFs are produced by Aspergillus spp, mainly Aspergillus flavus, Aspergillus parasiticus, Aspergillus nomius and Aspergillus pseudotamarii (2). AFs are potent teratogens, mutagens and carcinogens, classified as Group 1 carcinogens by the International Agency for Research on Cancer, primarily affecting liver (3). However, a provisional maximum tolerated daily intake (PMTDI) of 1 ng AF/Kg body weight (bw)/ day may be used as a guidance value in the risk assessment of AF from food (4). 

AFs have been detected in a number of foods including figs, nuts (peanut, walnut, almond and pistachio), cereals (wheat, maize, barley and rice), cottonseed and oil products (5). Because of potential health hazards to humans, regulatory levels have recently been documented. Currently, worldwide range of limits for aflatoxin B_1_ (AFB_1_) and total AF (AFT) are 1-20 and 0-35 ng/g, respectively (6). In Iran, the maximum tolerated level (MTL) of AFB_1_ for rice, wheat and peanut is 5 ng/g (6-7).

The use of reversed-phase high-performance liquid chromatography (RP-HPLC) method with fluorescence detection after immunoaffinity column (IAC) clean-up is common for AFs analysis. However, the analysis is time-consuming and total run time is approximately in the range of 6.5-15 min (8-10).

There are little data on the natural occurrence of AFs in cereals and nuts in Iran (11-12). Furthermore, exposure assessment of Iranian population to AFB_1_ has not been performed yet. In this study, we investigated the presence of AFB_1_ in various foods collected from Tehran retail market using a specifically developed and validated HPLC method. The results were then used for the first time for exposure assessment of Tehran population to AFB_1_.

## Experimental

All reagents were of analytical grade. Solvents used for the experiments were of either HPLC or analytical grade. The standard of AFB_1_ was purchased from Sigma-Aldrich. The IAC for AFB_1_ was purchased from Vicam Company, MA, USA. The chromatographic apparatus consisted of a Wellchrom K-1001 pump, a Rheodyne Model 7125 injector and a RF10AXL fluorescence detector connected to a Eurochrom 2000 integrator, all from Knauer (Berlin, Germany). The separation was performed on Chromolith Performance (RP-18e, 100 × 4.6 mm) column from Merck (Darmstadt, Germany).

*Sampling and sample preparation*

Samples (rice, bread, peanut, puffed corn snack and wheat flour) were collected in June 2005 by trained personnel from various sales outlets in nine geographic zones in Tehran, Iran, according to the sampling plan for official control of mycotoxins in food (13). All samples were finely ground by mill and subsamples were stored in freezer at - 32ºC until analysis.

*Preparation of AFB1 standard*

Stock, intermediate and working standard solutions of AFB1 were prepared according to Stroka *et al. *method (14). After the preparation of standard solution of AFB1 (10 μg/mL), the concentration was determined using UV spectrophotometer. This standard was used to prepare working standards of AFB1 for HPLC analysis (14).

*Aflatoxin analysis*

The method used for AFB1 extraction from samples and the chromatographic conditions were based on the AOAC official method 999.07 with some minor modifications (14). Briefly, fifty grams of samples including rice, bread, wheat flour, peanut and puffed corn snack was shaken for 30 min with 5 g of sodium chloride and 300 mL of methanol:H_2_O (80:20 v/v). For peanut and puffed corn snack, 100 mL of *n*-hexane was added too. After filtration, 20 mL of the filtrate was diluted with 130 mL of deionized water and filtered through the glass microfiber filter, and 75 mL of the filtrate was used for further clean up on a Afla test IAC column. The Afla test column was preconditioned with 10 mL of phosphate buffered saline (2-3 mL/min) and then 75 mL of the diluted sample extract was passed through the column (2-3 mL/min). Finally, the column was washed with 15 mL water. For AFB1 elution from the column, a portion of 0.5 mL HPLC grade methanol was passed through the column followed by an additional portion of 0.75 mL of the same solvent one minute afterward. HPLC grade water was added to the eluent to the volume of 3 mL and 100 μL of the final solution was injected into HPLC.

For all samples, separation was performed on a monolithic column (100 × 4.6 mm) using a HPLC system equipped with a fluorescence detector. Mobile phase was water: methanol (55:45, v/v) with a flow rate of 1.2 mL/min. The fluorescence detector was operated at excitation wavelength of 365 nm and emission wavelength of 435 nm. Post-column derivatization was carried out with pyridinium hydrobromide perbromide (PBPB) at flow rate of 0.4 mL/min.

*Method validation*

To evaluate the reliability of the results, in addition to apply regular validation assessment to the developed method, internal quality control experiments were also performed. In each working day, a blank and a spiked sample were also analyzed. According to the recovery values, AFB1 levels were corrected for recoveries. In addition, a certified reference material (CRM) from FAPAS (UK) was analyzed.

## Results and Discussion

*Method validation*

**Figure 1:**
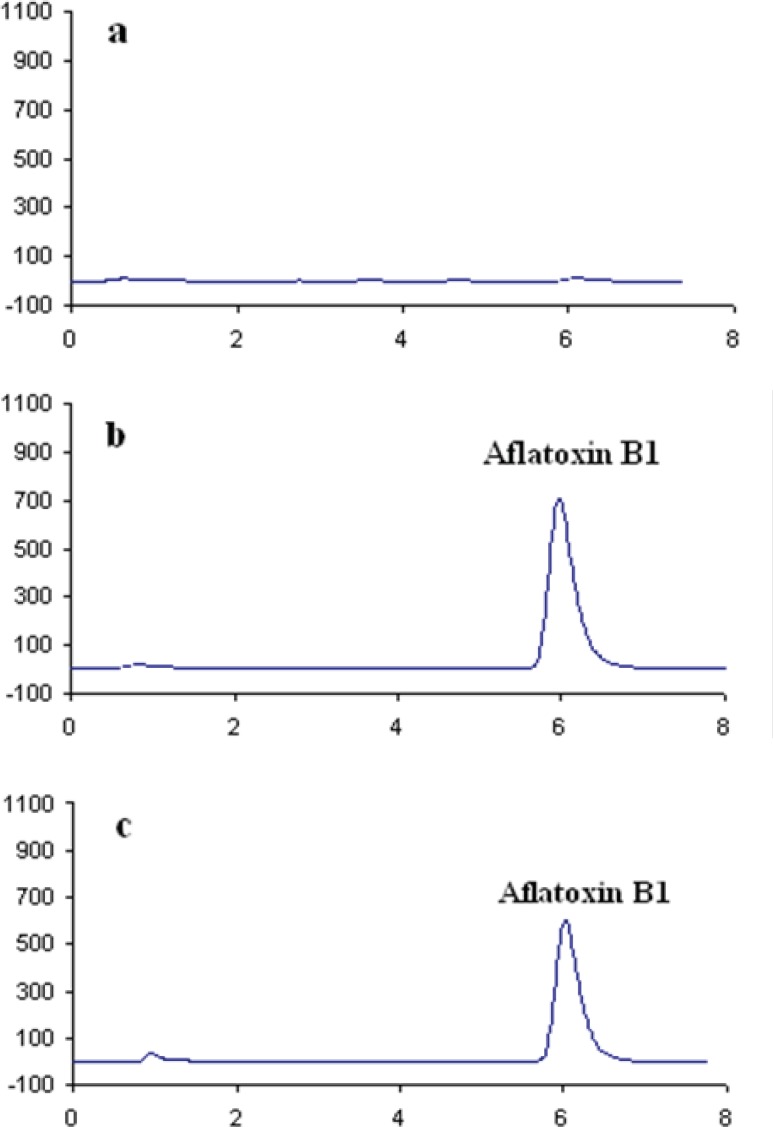
HPLC chromatograms of a: Blank rice sample, b: AFB1 standard (3.6 ng/mL) and c: Contaminated puffed corn snack (5 ng/g).

In order to perform the study on AFB_1_ contamination level in the five sample matrices, a specific high performance liquid chromatographic (HPLC) method was developed. Then, the method was validated in terms of linearity, limit of detection (LOD) and limit of quantification (LOQ), selectivity, precision and recovery. The method was satisfactory in terms of selectivity as an IAC was applied for purification of AFB_1_ which eliminated false positive results caused by interfering materials. Typical chromatograms obtained for AFB_1_ are shown in Figure 1.

Linearity was assessed for AFB_1_ over a range of 0.4-3.6 ng/g and reasonable correlation coefficients (r^2^ > 0.995) were obtained which indicated a good linearity of the analytical response over the specified concentration range. The estimated LOD (signal to noise ratio = 3) and LOQ (signal to noise ratio = 9) of AFB_1_ were 0.01 ng/g and 0.03 ng/g, respectively, which indicated that applying monolithic column improved sensitivity of the method compared to the previously reported methods (15-17).

Accuracy of the method was assessed by performing recovery experiments. Each test was performed three times and the average recoveries and relative standard deviation for repeatability (RSDr) are shown in Table 1. 

**Table 1:** Results of validation assessment of HPLC method developed for determination of AFB1 in different foods (n = 3).

**Sample**	**Spiking level (ng/g)**	**Recovery (%)**	**RSD**_r _ **(%)**
**Rice**	2	105.4	9.4
	5	97.8	6
	10	104.4	3.9
	Mean recovery ± SD	102.5 ± 4.13	4.03
**Bread**	2	99.9	13.4
	5	90.1	4.7
	10	109.8	13.2
	Mean recovery ± SD	99.9 ± 9.85	9.86
**Puffed corn snack**	2	101.8	4.8
	5	102.1	10.4
	10	100.2	9.6
	Mean recovery ± SD	101.4 ± 1.02	1.0
**Peanuts**	2	102.6	17.8
	5	98.2	12.6
	10	98.1	8.2
	Mean recovery ± SD	99.6 ± 2.57	2.58
**Wheat flour**	2	91.2	7.00
	5	94.7	7.4
	10	97.3	3.7
	Mean recovery ± SD	94.4 ± 3.06	3.24

The average recoveries (and RSDr) of AFB_1_ from spiked rice, bread, puffed corn snack, peanuts and wheat flour were 102.5% (4.03%), 99.9 (9.86%), 101.4% (1.0%), 99.6% (2.58%) and 94.4% (3.24%), respectively. These values fall well within the EU method performance criteria for AF analysis (13). The amount of AFB1 in corn CRM was 2.8 ng/g which is in the acceptable range of FAPAS Scheme (1.94-4.99 ng/g) and confirmed the accuracy of the analytical method.

**Table 2:** Natural occurrence of AFB1 in rice, bread, puffed corn snack, peanut and wheat flour samples marketed in Tehran, June 2005.

Samples	**Numbers of samples in the range (ng/g)**			
	< 0.03^a^	≥ 0.03 - < 2	≥ 2 - < 5	≥ 5
Rice	9	6	2	1
Bread	18	-	-	-
Puffed corn snack	7	11	-	-
Peanuts	4	10	2	2
Wheat flour	18	-	-	-

^a^ LOQ: 0.03 ng/g.

In the previous studies, the reported total run time for AFB_1_ was in the range of 6.5-15 min (8-10, 18). Applying monolithic column in the present work resulted in shorter analysis time (6 min) which is even faster than other published methods. Due to the application of monolithic column, less amount of organic phase was used, compared to the published methods, to optimize the resolution (19). Besides, in the present work, a mobile phase consisted of methanol:water was used, which resulted in omitting the more toxic and more expensive solvent, acetonitrile. Thus, our proposed HPLC method proved to be well suited for routine determination of AFB_1_ in the specified samples being around 10 samples processed per day.

*Application of method to the real samples*

The natural occurrence data for AFB_1_ in retail foods are shown in Tables 2 and 3.

**Table 3:** Contamination data for AFB1 (ng/g) in rice, bread, puffed corn snack, wheat flour and peanut samples marketed in Tehran, June 2005.

**Sample**	**No. of samples**	**Positive samples (%)**	**Mean** ^a^ **(± SD)**	**Median**	**Max**
**Rice**	18	9 (50)	4.17 (9.36 ±)	1.17	30.63
**Bread**	18	0	<LOQ	-	-
**Puffed corn snack**	18	11 (66.6)	0.11 (0.11 ±)	0.09	0.43
**Peanut**	18	14 (77.8)	1.97 (2.94±)	0.95	9.95
**Wheat flour**	18	0	<LOQ	-	-

a Mean of positive samples.

**Table 4:** Estimated daily intake of aflatoxin B1 (ng/g) through rice, bread, puffed corn snack and peanut samples marketed in Tehran, June 2005.

**Samples**	**Mean ** ^a, b^	**Max**	**Daily intake** **(ng /Kg bw** ^c^ **/day)**			
			**Mean**	**90th percentile**	**95th percentile**	**Max**
**Rice**	2.29	30.63	3.49	4.07	12.12	46.82
**Bread**	NA**d**	NA	NA	NA	NA	NA
**Puffed corn snack ** ^e^	0.12	0.43	0.11	0.19	0.46	0.43
**Peanuts**	1.54	9.95	0.02	0.05	0.11	0.14
**Total**	-	-	3.62	4.31	12.69	47.39

^a^: Mean of all samples; ^b^: The not detected values were replaced by half the limit of quantification; ^c^: Body weight (bw) for adults is assumed 70 Kg; ^d^: NA: Not applicable; ^e^: Mean weight of 65 g is assumed for each package

Our results showed that AFB_1 _was present in 34 out of 90 samples (37.7%). It is also shown that none of the bread and wheat flour samples contained detectable amounts of AFB_1_. The maximum amount of AFB_1_ detected in rice, puffed corn snack and peanut were 30.63, 0.43 and 9.95 ng/g, respectively, whereas the mean positive contamination levels of AFB_1_ were 4.17, 0.11, and 1.97 ng/g, in rice, puffed corn snack and peanut, respectively. The level of contamination of 3 samples (one rice sample and two peanuts samples) exceeded than Iranian MTL of 5 ng/g. Khamiri *et al. *reported that among 156 peanut samples analyzed for AFB_1_, one sample (0.64%) was contaminated at the level of 491 ng/g (11). Magrine *et al. *determined the concentrations of AFs in 100 samples of peanut products in Brazil. There was a 50% occurrence of AFs (B_1_, B_2_, G_1_ and G_2_) in concentrations ranging from 0.5 to 113 ng/g, with 13 samples with levels above 20 ng/g (20). In our study, 11.1% of peanut samples were contaminated at the levels higher than Iranian MTL.

There is only one report on AFB_1 _contamination in rice in Iran (12). Mazaheri reported that AFB_1_ and AFT were detected in 59 of 71 rice samples. The mean of AFB_1_ and AFT were 1.89 and 2.09 ng/g, respectively (12). Our result is in agreement with Mazaheri study and in both studies, the mean of AFB_1_ were lower than Iranian MTL. In Austria, a survey has been carried out on the natural occurrence of AFB1 in rice. AFB1 was found in 24 out of 81 samples. The contamination range was between 0.45 ng/g and 9.86 ng/g. Three samples exceeded the maximum levels set in the European Union; having AFB_1_ concentrations of 2.16, 2.85 and 9.86 ng/g (21). In our study, just one sample exceeded the Iranian MTL.

Abdullah *et al*. analyzed 83 wheat flour samples in Malaysia and reported that 1.2% of samples were positive for AFB_1_, at a concentration of 25.6 μg/Kg (22). In our study, none of the wheat flour samples contained detectable amounts of AFB_1_.

*Estimation of dietary intake of AFB*_1_

This is the first study on exposure assessment of Iranian population to AFB_1_. Exposure to mycotoxins for each type of food depends on the mycotoxins concentration in food and the amount of food consumed. In this study, the consumption rates of rice and bread were based on a consumption survey performed in Iran since 2001-2003 (23*)*. The average consumption of rice and bread are 107 g and 286 g per day per person in Tehran, respectively. Unfortunately, there are no data of puffed corn snack and peanut consumption in Iran so we assumed a package (65 g) per day for puffed corn snack and 1 g per day for peanut as mean daily consumption for the two products.

Estimated daily intakes of AFB_1_ in different foods are given in Table 4.

The tolerable daily intake for genotoxic compounds (such as AFB_1_) cannot be used as a safety factor, since the intake of such substances should be kept as low as reasonably achievable. However, WHO has suggested that a PMTDI of 1 ng AF/Kg bw/day may be used as a guidance value in the risk assessment of AF from food (4). Although mean estimated daily intake of AFB_1_ from all analyzed foods was 3.62 ng/Kg bw/day, the mean dietary intake of AFB_1_ from rice alone was estimated 3.49 ng/Kg bw/day which is ca 3.5 times higher than the guidance value of 1 ng AF/Kg bw/day (Table 4). Considering the high consumption rates of rice and bread in Iran, it is recommended to reduce the MTL of AFs in these foods.

The estimated probable daily intake for AFB_1_ in samples of peanut products analyzed in Brazil varied from 0.6 to 10.4 ng/Kg bw/day (20). In South Korea, among 88 rice samples, AFB_1_ was detected in 5/88 (6%) of samples with an average of 4.8 ng/g. A calculated probable daily intake of AFB_1_ for Koreans fell into the range of 1.19-5.79 ng/Kg bw/day (24).

## Conclusion

The specifically developed HPLC method was found to be accurate, sensitive, safer and more economic, and meets EU method performance criteria for AFB_1_ analysis. Compared to other reported methods, it is faster and results in a remarkably low LOD of 0.01 ng/g. This method is suitable for routine analysis of AFB_1_. Upon applying the proposed method, in most of the samples the level of AFB_1_ was found to be lower than the Iranian MTL. However, due to the high consumption of foods like rice, the risk of exposure to AFB_1_ is higher than the guidance value of 1 ng/Kg bw/day and requires closer monitoring of the contamination in foods.

## Notes

There is no conflict of interests.
